# Evaluation of the Radiometer ‘ABL4’ pH/blood gas/K^+^/Hb analyser

**DOI:** 10.1155/S146392468900043X

**Published:** 1989

**Authors:** Bénédicte Beneteau-Burnat, Michel Vaubourdolle, Messereth Kebede, Richard Kowalezyk, Jacqueline Giboudeau

**Affiliations:** Laboratoire de Biochimie A Hôpital Saint Antoine 184, rue du Fg Saint Antoine Paris Cedex 12 75571 France


Journal of Automatic Chemistry Vol. 11, No. 5 (September–October 1989), pp. 221–222

### Evaluation of the Radiometer 'ABL4' pH/blood gas/$K^+$/Hb analyser

**Bénédicte Beneteau-Burnat, Michel Vaubourdolle, Messereth Kebede, Richard Kowalezyk and Jacqueline Giboudeau**

*Laboratoire de Biochimie A, Hôpital Saint Antoine, 184, rue du Fg Saint Antoine, 75571 Paris Cedex 12, France*

The ABL4 analyser is designed for stat determination of blood gas ($pCO_2$, $pO_2$), pH, potassium and haemoglobin (Hb), a panel particularly adapted to the follow-up of liver transplantation. The performance of the analyser in classical tests was evaluated; in addition, $K^+$ measurements were performed in specific conditions (linearity in a non-physiological range, erythrocyte interference).

The evaluation of precision and comparison of methods were carried out according to the guidelines of the SFBC (Société Française de Biologie Clinique), which are similar to those of the NCCLS (EP5-P and EP9-P). Within-run and between-run precision and percentage CV were within the allowable limits of error (see table 1).

The results obtained with the ABL4 correlated well with those from previously validated analysers (table 2). It should be noted that the slope for Hb can be adjusted to 1, using a special built-in function of the ABL4.

*Table 1. Precision.*

| Analyte | Level | Within-run ($N$ = 30) Mean | SD | CV % | Between-run ($N$ = 20) Mean | SD | CV% |
|---|---|---|---|---|---|---|---|
| | L | 7·099 | 0·002 | 0·03 | 7·112 | 0·005 | 0·1 |
| pH | M | 7·356 | 0·0036 | 0·05 | 7·359 | 0·005 | 0·1 |
| | H | 7·590 | 0·0025 | 0·03 | 7·595 | 0·004 | 0·1 |
| | L | 20·5 | 0·24 | 1·2 | 19·8 | 0·6 | 2·8 |
| $pCO_2$ | M | 42·2 | 0·61 | 1·4 | 41·9 | 0·9 | 2·3 |
| (mmHg) | H | 63·5 | 0·35 | 0·5 | 62·2 | 1·8 | 3·0 |
| | L | 50·2 | 0·9 | 1·8 | 49·3 | 1·6 | 3·3 |
| $pO_2$ | M | 108·4 | 1·3 | 1·2 | 104·1 | 2·6 | 2·5 |
| (mmHg) | H | 185·6 | 1·8 | 0·95 | 176·2 | 2·9 | 1·6 |
| | L | 2·49 | 0·03 | 1·2 | 2·4 | 0·1 | 2·4 |
| $K^+$ conc | M | 4·01 | 0·03 | 0·75 | 3·9 | 0·1 | 1·8 |
| (mmol/l) | H | 5·19 | 0·03 | 0·58 | 5·0 | 0·1 | 2·7 |
| | L | 6·85 | 0·15 | 2·1 | 6·80 | 0·22 | 3·3 |
| Hb conc | M | 14·58 | 0·13 | 0·9 | 14·45 | 0·35 | 2·4 |
| (g/l) | H | 20·00 | 0·07 | 0·3 | 20·20 | 0·30 | 1·5 |

Concentration levels: L = low, M = medium, H = high.

*Table 2. Comparison of methods (N = 100).*

| Analyte | Reference analyser | Slope | Intercept | $r$ |
|---|---|---|---|---|
| pH | Corning 178 (potentiometry) | 0·996 | + 0·02 | 0·995* |
| $pCO_2$ | Corning 178 (potentiometry) | 0·990 | − 0·23 | 0·987* |
| $pO_2$ | Corning 178 (polarography) | 1·066 | − 2·8 | 0·996* |
| $K^+$ | Corning 634 (direct potentiometry) | 1·061 | − 0·35 | 0·986* |
| Hb | Minos St (Drabkin method) | 0·897 | + 0·44 | 0·954* |

*$p$ <0·001

0142–0453/89 $3.00 © 1989 Taylor & Francis Ltd.

Concerning $K^+$, a linear response up to 55 mM potassium was demonstrated in the lavage solution used in liver transplantation. In emergencies, this large linearity range avoids the time-consuming dilution step required with some other whole-blood $K^+$ analysers. Furthermore, there was no difference between plasma (centrifuged at 4°C) and whole-blood $K^+$ determinations (mean difference = 0·07, $N = 100$). The absence of RBC interference is important during prolonged surgical interventions which are associated with changes in haematocrit values.

In conclusion, the ABL4 is a reliable blood-gas analyser. In addition, whole-blood $K^+$/Hb determinations are good, making the equipment suitable for the management of liver transplantation.

### NOTES FOR AUTHORS

*Journal of Automatic Chemistry* covers all aspects of automation and mechanization in analytical, clinical and industrial environments. The *Journal* publishes original research papers; short communications on innovations, techniques and instrumentation, or current research in progress; reports on recent commercial developments; and meeting reports, book reviews and information on forthcoming events. All research papers are refereed.

### Manuscripts

Two copies of articles should be submitted. All articles should be typed in double spacing with ample margins, on one side of the paper only. The following items should be sent: (1) a title-page including a brief and informative title, avoiding the word 'new' and its synonyms; a full list of authors with their affiliations and full addresses; 12) an abstract of about 250 words; (3) the main text; (4) appendices (if any); (5) references; (6) tables, each table on a separate sheet and accompanied by a caption; (7) illustrations (diagrams, drawings and photographs) numbered in a single sequence from 1 upwards and with the author's name on the back of every illustration; captions to illustrations should be typed on a separate sheet. Papers are accepted for publication on condition that they have been submitted only to this *Journal.*

### References

References should be indicated in the text by numbers following the author's name, i.e. Skeggs [6]. In the reference section they should be arranged thus:

to a journal

Manks, D. P., *Journal of Automatic Chemistry*, **3** (1981), 119.

to a book

Malmstadt, H. V., in *Topics in Automatic Chemistry*, Ed. Stockwell, P. B. and Foreman, J. K. (Horwood, Chichester, 1978), p. 68.

### Illustrations

Original copies of diagrams and drawings should be supplied, and should be drawn to be suitable for reduction to the page or column width of the *Journal*, i.e. to 85 mm or 179 mm, with special attention to lettering size. Photographs may be sent as glossy prints or as negatives.

### Proofs and offprints

The principal or corresponding author will be sent proofs for checking and will receive 50 offprints free of charge. Additional offprints may be ordered on a form which accompanies the proofs.

*Manuscripts should be sent to Dr P. B. Stockwell, P.S. Analytical Ltd, Arthur House, Unit B4, Chaucer Business Park, Watery Lane, Kemsing, Sevenoaks, Kent TN1S 6QY, UK.*

